# Clinical Characteristics and Predictors of One-Year Mortality in HIV and Non-HIV Patients with Cryptococcal Infections in a Middle-Income Setting: A Multicenter Cohort Study

**DOI:** 10.3390/jof12080545

**Published:** 2026-07-23

**Authors:** Aysun Benli, Atahan Çağatay, Berivan Şahin, Tuğba Arslan Gülen, Tuba Turunç, Emine Günal, Özlem Güler, Esra Kazak, Ferit Kuşcu, Ayça Aydın, Zeynep Karakaşoğlu, Neşe Saltoğlu, Süheyla Serin Senger, Hale Turan Özden, Buket Erturk Sengel, Recep Tekin, Oğuz Usta, Esra Gürbüz, Zehra Çağla Karakoç, Didem Akal Taşcıoğlu, İlkay Karaoğlan, Yasemin Tezer

**Affiliations:** 1 Department of Infectious Diseases and Clinical Microbiology, İstanbul Faculty of Medicine, İstanbul University, İstanbul 34093, Türkiye; 2 Department of Infectious Diseases and Clinical Microbiology, Adana City Training and Research Hospital, University of Health Sciences, Adana 01230, Türkiye; 3 Department of Infectious Diseases and Clinical Microbiology, İstanbul Prof. Dr. Cemil Taşcıoğlu City Hospital, İstanbul 34384, Türkiye; 4 Department of Infectious Diseases and Clinical Microbiology, Faculty of Medicine, Kocaeli University, Kocaeli 41380, Türkiye; 5 Department of Infectious Diseases and Clinical Microbiology, Faculty of Medicine, Bursa Uludağ University, Bursa 16059, Türkiye; 6 Department of Infectious Diseases and Clinical Microbiology, Faculty of Medicine, Çukurova University, Adana 01330, Türkiye; 7 Department of Infectious Diseases and Clinical Microbiology, Sultan 2. Abdulhamid Han Training and Research Hospital, University of Health Sciences, İstanbul 34668, Türkiye; 8 Department of Infectious Diseases and Clinical Microbiology, Faculty of Medicine, İstanbul University-Cerrahpaşa, İstanbul 34098, Türkiye; 9 Department of Infectious Diseases and Clinical Microbiology, İzmir Tepecik Training and Research Hospital, University of Health Sciences, İzmir 35020, Türkiye; 10 Department of Infectious Diseases and Clinical Microbiology, Marmara University Pendik Training and Research Hospital, İstanbul 34899, Türkiye; 11 Department of Infectious Diseases and Clinical Microbiology, Faculty of Medicine, Dicle University, Diyarbakır 21280, Türkiye; 12 Department of Infectious Diseases and Clinical Microbiology, Faculty of Medicine, Koç University, İstanbul 34010, Türkiye; 13 Department of Infectious Diseases and Clinical Microbiology, Van Training and Research Hospital, University of Health Sciences, Van 65300, Türkiye; 14 Department of Infectious Diseases and Clinical Microbiology, Faculty of Medicine, İstinye University, İstanbul 34517, Türkiye; 15 Department of Infectious Diseases and Clinical Microbiology, Faculty of Medicine, Gaziantep University, Gaziantep 27850, Türkiye; 16 Department of Infectious Diseases and Clinical Microbiology, Ankara Bilkent City Hospital, Ankara 06800, Türkiye

**Keywords:** *Cryptococcus neoformans*, *Papiliotrema laurentii*, cryptococcal infections, mortality, prognostic factors, HIV, PLWH, immunocompromised host, albumin, central nervous system

## Abstract

Background: Cryptococcal infections remain life-threatening fungal infections affecting both people living with HIV (PLWH) and non-HIV populations. Data from real-world cohorts in middle-income settings, particularly those including both groups, remain limited. This study aimed to evaluate the clinical characteristics and identify predictors of one-year mortality in patients with cryptococcal infections. Methods: In this retrospective multicenter cohort study, adult patients diagnosed with cryptococcal infection between 2013 and 2025 were included and followed for one year after diagnosis or until death. Demographic, clinical, laboratory, and treatment data were collected. Survival was analyzed using Kaplan–Meier methods and compared with the log-rank test. Cox proportional hazards regression was used to identify factors independently associated with mortality. Subgroup analyses were performed according to HIV status. Results: A total of 92 patients with an average age of 46 ± 16 years were included; 56.5% were PLWH, and 87% were immunocompromised. Central nervous system involvement was the most common presentation (64.1%). Overall one-year mortality was 46.7%. In the multivariable Cox regression analysis, altered consciousness (HR 3.110, 95% CI 1.624–5.956, *p* < 0.001) and disseminated infection (HR 2.155, 95% CI 1.156–4.016, *p* = 0.016) were independently associated with increased mortality, whereas higher serum albumin levels were independently associated with improved survival (HR 0.532, 95% CI 0.328–0.861, *p* = 0.010). Age remained independently associated with mortality (HR 1.025 per year, 95% CI 1.006–1.045, *p* = 0.010). Kaplan–Meier analysis showed significantly lower one-year survival among patients with altered consciousness and disseminated infection. Although CNS involvement was more frequent among PLWH (78.8% vs. 45%, *p* < 0.001), mortality rates were comparable between PLWH and non-HIV patients. Conclusions: Cryptococcal infections are associated with high mortality regardless of HIV status. Altered consciousness and disseminated infection were independently associated with one-year mortality, whereas higher albumin levels were associated with improved survival. Simple clinical parameters may contribute to early clinical risk assessment, particularly in resource-limited settings.

## 1. Introduction

The majority of human cryptococcal infections are caused by members of the *Cryptococcus neoformans*/*C. gattii* species complex. Rare opportunistic infections caused by other encapsulated basidiomycetous yeasts, such as *Papiliotrema laurentii* (formerly *Cryptococcus laurentii*), have also been reported, particularly in immunocompromised (IC) individuals [1,2]. Cryptococcal infection is caused by an encapsulated yeast that can lead to meningoencephalitis, pneumonia, and skin infections in IC individuals, particularly in people living with HIV (PLWH). It remains one of the leading causes of fungal-related mortality worldwide, particularly among PLWH [3]. Despite improvements in antifungal therapy and antiretroviral treatment, cryptococcal meningitis continues to account for substantial global mortality, especially in resource-limited settings [4]. Globally, there are approximately 1 million cases and more than 600,000 deaths annually. Although traditionally associated with advanced HIV infection, cryptococcosis increasingly affects non-HIV populations, including patients with hematological malignancies, solid organ transplantation, autoimmune diseases requiring immunosuppressive therapy, cirrhosis, diabetes mellitus, and even individuals without identifiable immunodeficiency [5,6,7]. Several multicenter cohorts have demonstrated that non-HIV, non-transplant patients may experience equal or even higher mortality compared to PLWH [5,6]. This condition underscores that immune competence does not universally confer protection and may mask disease severity until late.

Reported mortality rates in cryptococcal meningitis or disseminated cryptococcosis range between 20% and 50%, depending on host immune status, central nervous system (CNS) involvement, fungal burden, and the adequacy of antifungal therapy [3,8]. Altered mental status, hydrocephalus, elevated intracranial pressure, dissemination, and delayed treatment initiation have consistently been associated with poor outcomes [6,9]. Adequate induction therapy with liposomal amphotericin B (L-AmB)-based regimens significantly improves survival [10,11]. International guidelines and the World Health Organization emphasize L-AmB plus flucytosine as first-line induction therapy and highlight the importance of aggressive intracranial pressure management [10]. The duration of induction therapy has also been linked to mortality risk [11]. In addition to clinical factors, laboratory biomarkers such as the neutrophil-to-lymphocyte ratio (NLR), lymphopenia, and inflammatory markers have been explored as prognostic indicators in cryptococcosis [7,12]. The ASM/ECMM/ISHAM global guideline underscores the clinical complexity of cryptococcosis and the need for precise risk stratification to guide timely diagnostic and therapeutic decisions [13].

A number of studies have been conducted that have identified poor prognostic factors in cryptococcal infections. In Türkiye, only one study has been conducted in this context in recent years, by Hakyemez et al. [14]. However, given that the study incorporates patient data from multiple countries, the findings do not accurately reflect the demographics of our geographical region. Despite extensive data from high-income settings, evidence from middle-income countries remains limited, particularly regarding treatment constraints such as restricted access to flucytosine and their impact on outcomes. Furthermore, the epidemiological shift toward non-HIV populations necessitates updated comparative analyses across different immune status groups. In Türkiye, multicenter data evaluating prognostic factors in cryptococcal infections are scarce. Therefore, this study aimed to analyze a nationwide multicenter cohort of patients with cryptococcal infections, identify factors independently associated with mortality, and compare the clinical characteristics and outcomes between PLWH and non-HIV patients.

## 2. Materials and Methods

### 2.1. Study Design and Setting

This retrospective multicenter cohort study was conducted across 16 tertiary hospitals in Türkiye. Patients followed by physicians who were members of the Turkish Clinical Microbiology and Infectious Diseases Association (KLİMİK) Fungal Study Group (MİÇG) were included in the study. Each center contributed data using a standardized electronic case report form developed by the coordinating investigator. The study protocol was approved by the institutional review boards of the participating centers. The primary outcome was one-year all-cause mortality. All patients were followed for a minimum of 365 days after diagnosis. For patients who died within the first year, follow-up ended on the date of death.

### 2.2. Participants

Adult patients (age ≥ 18 years) diagnosed with cryptococcal infection between January 2013 and June 2025 were included in the study. Diagnosis required clinical findings consistent with cryptococcal infection and at least one of the following: 1. Positive culture for *Cryptococcus* spp. from sterile sites. 2. Positive cryptococcal antigen (CrAg) detection in serum or cerebrospinal fluid (CSF). 3. Histopathological evidence consistent with cryptococcal infection. Patients were excluded if clinical data were incomplete or if microbiological confirmation was lacking.

### 2.3. Data Collection

Data were retrospectively extracted from electronic medical records or patients’ files using a standardized data collection form. The following variables were recorded: demographics (age and sex), comorbidities and immunosuppression status, duration of immunosuppression, clinical presentation (fever, headache, seizure, altered consciousness, etc.), Glasgow Coma Scale (GCS) score, infection involvement type (CNS, pulmonary, skin, and disseminated), laboratory parameters (complete blood count, neutrophil-to-lymphocyte ratio (NLR), C-reactive protein (CRP), erythrocyte sedimentation rate (ESR), and albumin), fungal culture results, serum/CSF cryptococcal antigen results, radiological findings, intensive care unit (ICU) admission, and outcomes (mortality, relapse, and sequelae). Antifungal therapy details and adjunctive interventions (lumbar punctures to manage intracranial pressure) were also documented.

### 2.4. Definitions

CNS involvement was defined as the presence of clinical features consistent with meningitis or meningoencephalitis, along with either abnormal CSF findings or neuroimaging compatible with cryptococcal infection. Altered consciousness was defined using the Glasgow Coma Scale (GCS) recorded at presentation. A GCS score < 15 was considered indicative of altered consciousness in the primary analysis, as this threshold was intended to capture any degree of impaired consciousness. To evaluate the consistency of the findings, a sensitivity analysis was also performed using a GCS score ≤ 13, a commonly accepted threshold for moderate impairment of consciousness. Pulmonary involvement was defined as radiological findings suggestive of pulmonary infection combined with microbiological confirmation from respiratory samples. Cutaneous involvement was defined as skin lesions with microbiological or histopathological confirmation of *Cryptococcus* spp. Disseminated infection was defined as involvement of two or more non-contiguous organ systems and/or positive blood cultures [1]. Immunosuppression included HIV infection, malignancy, solid organ transplantation, immunosuppressive therapy, or other conditions associated with impaired immunity. Relapse was defined as the recurrence of clinical disease with microbiological confirmation after initial improvement. Sequelae were defined as persistent neurological deficits at the last follow-up visit. Immune reconstitution inflammatory syndrome (IRIS) was defined as paradoxical clinical worsening after the initiation of antiretroviral therapy in PLWH [15]. Induction therapy was defined as the initial antifungal treatment phase, typically including L-AmB-based regimens with flucytosine. L-AmB was combined with fluconazole in the absence of flucytosine.

### 2.5. Laboratory Work

Clinical specimens were processed in the local microbiology laboratories according to the routine diagnostic procedures of each participating center. Where available, isolates were identified by matrix-assisted laser desorption/ionization time-of-flight mass spectrometry (MALDI-TOF MS). In participating laboratories without MALDI-TOF MS, conventional phenotypic methods, including standard biochemical tests and canavanine-glycine-bromothymol blue (CGB) agar, were used for species identification and differentiation of the *Cryptococcus neoformans*/*C. gattii* species complex. *Papiliotrema laurentii* (formerly *Cryptococcus laurentii*) isolates were identified by MALDI-TOF MS. No isolates belonging to *C. gattii* were identified during the study period. Cryptococcal antigen (CrAg) testing in serum and/or cerebrospinal fluid was performed using either latex agglutination or a lateral flow assay, according to institutional availability.

### 2.6. Statistical Analysis

Statistical analyses were performed using the Statistical Package for the Social Sciences (SPSS) v.28.0 (IBM Corp., Armonk, NY, USA). Categorical variables were presented as frequencies and percentages, while continuous variables were expressed as mean ± standard deviation (SD). Comparisons between groups were performed using the Mann–Whitney U test or Student’s *t*-test for continuous variables and Fisher’s exact test or chi-square test for categorical variables, as appropriate. Overall survival was defined as the time from the date of cryptococcal infection diagnosis to death from any cause or the end of the one-year follow-up period. Patients who survived beyond one year were censored at 365 days. Survival probabilities were estimated using the Kaplan–Meier method and compared using the log-rank test. To identify factors associated with one-year mortality, univariable Cox proportional hazards regression analyses were initially performed for each potential predictor. Variables with a *p* value < 0.10 in the univariable analyses, together with clinically relevant variables, were considered for inclusion in the multivariable Cox proportional hazards model. The final multivariable model was fitted using the enter method. Analyses were performed using complete-case data; no imputation was performed. The proportional hazards assumption was assessed graphically using log-minus-log survival plots, and no major violations were observed. Given the limited number of events, the number of variables included in the multivariable model was restricted to reduce the risk of overfitting. Hazard ratios (HRs) with 95% confidence intervals (CIs) were calculated. A two-sided *p* value < 0.05 was considered statistically significant.

## 3. Results

### 3.1. Patient Characteristics

A total of 92 patients with cryptococcal infection were included in the study. The mean age was 46 ± 16 years, and 72% were male. The majority of patients (87%) were IC, with 56.5% being PLWH. The mean duration of immunosuppression prior to diagnosis was 26 ± 37 months. The mean duration of symptoms until diagnosis was 50 ± 80 days. The most common presenting symptoms were fever (66.3%), headache (58.7%), altered consciousness (43.5%), nausea/vomiting (42.4%), and cough (41.3%). CNS involvement was the most frequent clinical manifestation (64.1%), followed by pulmonary involvement (28.3%) and disseminated infection (27.2%). Cutaneous involvement was observed in 12% of patients. *Cryptococcus neoformans* was the predominant species (66.3%), while *P. laurentii* accounted for 8.7% of cases. Overall mortality was 46.7%. Among survivors, relapse occurred in 7.6%, and neurological sequelae occurred in 13%. Of the 12 individuals without IC, 3 had chronic liver disease (2 of whom died), 3 had diabetes mellitus (1 of whom died), 1 had chronic lung disease, and 5 had no known disease.

### 3.2. Comparison Between Survivors and Non-Survivors

Comparisons between survivors (*n* = 49) and non-survivors (*n* = 43) are summarized in Table 1 and Table 2. Non-survivors were older (51 ± 18 vs. 42 ± 13 years, *p* = 0.007) and had a longer duration of immunosuppression (34 ± 42 vs. 18 ± 30 months, *p* = 0.001). The use of immunosuppressive agents was more frequent among non-survivors (41.8% vs. 22.4%, *p* = 0.046), although the overall prevalence of immunosuppression and HIV infection did not differ significantly between groups. Clinically, non-survivors more frequently presented with fever (79% vs. 55.1%, *p* = 0.015), altered consciousness (65.1% vs. 24.4%, *p* < 0.001), and seizures (27.9% vs. 8.1%, *p* = 0.013). They also had lower GCS scores at presentation (11.4 ± 3.4 vs. 14 ± 1.6, *p* < 0.001). Disseminated infection was more common among non-survivors (39.5% vs. 16.3%, *p* = 0.013), whereas cutaneous involvement was less frequent (4.6% vs. 18.3%, *p* = 0.043). Laboratory findings showed that non-survivors had higher inflammatory markers, including NLR (24.1 ± 34 vs. 7.8 ± 6.8, *p* = 0.004), CRP (67 ± 63 vs. 25 ± 41 mg/L, *p* < 0.001), and ESR (63 ± 29 vs. 46 ± 27 mm/h, *p* = 0.004). They also had lower lymphocyte counts, hemoglobin, platelet counts, and albumin levels (*p* < 0.05). With respect to treatment, the duration of L-AmB therapy was shorter among non-survivors (13 ± 8 vs. 26 ± 14 days, *p* < 0.001). Fluconazole use and duration were also lower in non-survivors (*p* = 0.002 and *p* < 0.001, respectively). Intensive care unit (ICU) admission was significantly more frequent among non-survivors (74.4% vs. 26.5%, *p* < 0.001).

### 3.3. Papiliotrema laurentii Characteristics

Eight patients (8.7%) had infections due to *P. laurentii,* of whom six were IC patients. The majority of these cases presented with pulmonary involvement (62.5%). Two patients died during follow-up. Given the small sample size, no formal statistical comparisons were performed for this subgroup. The baseline characteristics of patients with *P. laurentii* infections are presented in Table 3.

### 3.4. Multivariate Cox Regression Analysis for Mortality

**Figure 1:**
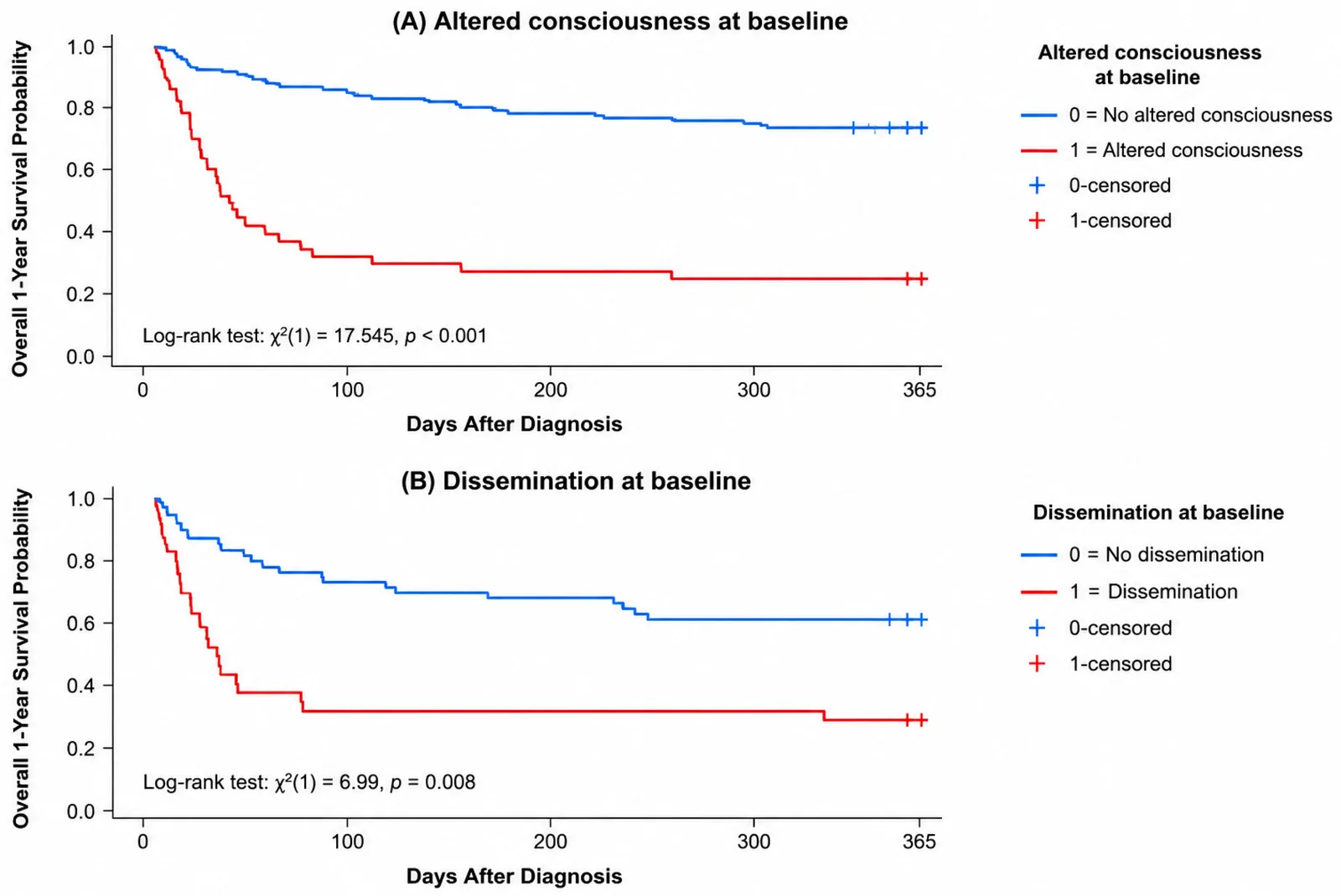
Kaplan–Meier curves for one-year overall survival stratified by altered consciousness (**A**) and dissemination (**B**) at presentation in patients with cryptococcal infection. Patients presenting with altered consciousness and those with disseminated infection had significantly lower one-year survival than patients without these features (log-rank test *p* < 0.001 and *p* = 0.008, respectively).

In univariate Cox regression analysis, age, disseminated infection, altered consciousness, and albumin levels were significantly associated with one-year mortality. In multivariable analysis, altered consciousness (HR 3.110, 95% CI 1.624–5.956, *p* < 0.001) and disseminated infection (HR 2.155, 95% CI 1.156–4.016, *p* = 0.016) were independently associated with increased mortality, while higher albumin levels were independently associated with improved survival (HR 0.532, 95% CI 0.328–0.861, *p* = 0.010). Age remained a modest but significant predictor of mortality (HR 1.025, 95% CI 1.006–1.045, *p* = 0.010) (Table 4). Kaplan–Meier curves for one-year overall survival stratified by altered consciousness and dissemination at presentation are shown in Figure 1. Patients presenting with altered consciousness had significantly worse one-year survival compared to those without (log-rank *p* < 0.001), with a median survival of 21 days. Likewise, dissemination at baseline was associated with significantly reduced one-year survival (log-rank test *p* = 0.008), with a median survival of 36 days.

### 3.5. Sensitivity Analyses

To evaluate the consistency of the primary findings, three sensitivity analyses were performed. First, patients with *P. laurentii* infection were excluded from the multivariable Cox regression analysis. The factors associated with one-year mortality remained consistent, with advanced age, disseminated infection, altered consciousness, and lower serum albumin continuing to be independently associated with mortality (Supplementary Table S1). Second, HIV status was forced into the multivariable Cox regression model because it was central to the study objective. HIV infection was not independently associated with one-year mortality (adjusted HR 0.865, 95% CI 0.436–1.716; *p* = 0.678), and its inclusion did not materially alter the effect estimates of the other variables (Supplementary Table S1). Third, a sensitivity analysis was performed using a GCS threshold of ≤13 instead of <15 to define altered consciousness. This analysis yielded similar results, with altered consciousness remaining significantly associated with one-year mortality in both univariable (HR 3.34, 95% CI 1.81–6.17; *p* < 0.001) and multivariable analyses (adjusted HR 2.81, 95% CI 1.50–5.27; *p* = 0.001), confirming the robustness of the findings. Overall, all sensitivity analyses yielded results consistent with the primary analysis, supporting the robustness of the study findings.

### 3.6. PLWH vs. Non-HIV Patients Analysis

Non-HIV patients were significantly older than PLWH (56 ± 17 vs. 41 ± 11 years, *p* = 0.002) and more frequently female (45% vs. 13.5%, *p* < 0.001). Headache and increased intracranial pressure were more frequently observed in PLWH (73.1% vs. 40%, *p* < 0.001 and 36.5% vs. 15%, *p* = 0.021, respectively), and CNS involvement was significantly higher in this group (78.8% vs. 45%, *p* < 0.001). Laboratory findings differed between groups, with non-HIV patients demonstrating higher leukocyte and neutrophil counts (both *p* < 0.001), whereas inflammatory markers such as ESR were higher in PLWH (*p* = 0.015). Serum and CSF cryptococcal antigen positivity rates were also higher in PLWH (*p* = 0.021 and *p* = 0.016, respectively). Regarding management, L-AmB was used more frequently in PLWH (88.5% vs. 70%, *p* = 0.027), and the hospital length of stay was longer in this group (47 ± 83 vs. 25 ± 29 days, *p* = 0.017). Non-HIV patients had longer ICU stays (23 ± 24 vs. 11 ± 11 days, *p* = 0.037), although ICU admission rates were comparable. Mortality was numerically higher in non-HIV patients (52.5% vs. 42.3%). However, this difference did not reach statistical significance (Supplementary Tables S2 and S3).

## 4. Discussion

To our knowledge, this represents one of the largest multicenter cohorts of patients with cryptococcal infections from Türkiye, encompassing both PLWH and non-HIV populations. Given regional differences in access to antifungal agents, healthcare organization, and epidemiological patterns, these findings provide real-world evidence from a middle-income setting. This multicenter study found that disseminated infection, altered consciousness, and advanced age were independently associated with one-year mortality in patients with cryptococcal infections. In addition, higher serum albumin levels were independently associated with improved survival.

The overall mortality rate of 46.7% observed in our cohort is consistent with previously reported global mortality rates, particularly in studies involving a high proportion of CNS involvement and advanced disease [3,16]. However, these data are largely derived from studies indicating high mortality rates in advanced stages of the disease in sub-Saharan Africa. Compared with multicenter cohorts from high-income settings, the mortality observed in our study appears higher, which may be explained by delayed diagnosis, prolonged symptom duration, and limited access to flucytosine [8,17]. Indeed, in a study in which the entire cohort consisted of IC hosts and the mean age was higher, the mortality rate was less than half that observed in the present study [18]. In another study, a shorter time to diagnosis was associated with an overall mortality rate of approximately 10% [19].

In various studies, malignancy, headache, advanced age, seizures, altered consciousness, low CD4+ T lymphocyte count, and diagnosis in previous years (2000 and earlier) were identified as factors predicting mortality in multivariate analyses [14,20,21,22]. Dissemination reflects an advanced stage of disease characterized by hematogenous spread and a high fungal burden, which has been consistently associated with poor outcomes in prior studies [23,24]. In our cohort, dissemination was independently associated with mortality, emphasizing the need for early detection of systemic involvement and aggressive treatment strategies.

Among the variables included in the multivariable analysis, altered consciousness was strongly associated with one-year mortality in our cohort, consistent with previous reports. It reflects advanced CNS involvement and severe disease burden. Kaplan–Meier analysis further supports this, showing markedly reduced survival among patients presenting with altered consciousness. We performed a sensitivity analysis using a GCS threshold of ≤13, a commonly accepted definition of moderate impairment of consciousness. The association between altered consciousness and one-year mortality remained statistically significant, indicating that our findings were robust to the choice of GCS cut-off. Cryptococcal meningoencephalitis often leads to increased intracranial pressure, cerebral edema, and neuronal dysfunction, all of which contribute to impaired consciousness [25,26]. This finding highlights the importance of early neurological assessment and timely management of intracranial pressure, including therapeutic lumbar punctures. This life-saving intervention is a vital component of treatment, particularly in cases of cryptococcal meningitis. Furthermore, some studies have indicated that intrathecal antifungal therapy can also be utilized as salvage therapy in certain cases, and intrathecal therapy has led to increased survival [27,28].

Advanced age was independently associated with mortality, likely reflecting the impact of immunosenescence. Aging-related impairments in innate immune function, including reduced phagocytosis and dysregulated inflammatory responses, may lead to inadequate fungal clearance and increased tissue damage. In addition, age-related disruption of the blood–brain barrier may facilitate central nervous system invasion, while atypical clinical presentations in older patients may delay diagnosis. Together, these mechanisms likely contribute to the poorer outcomes observed in elderly patients with cryptococcosis [29].

A particularly important finding was the association between a shorter duration of L-AmB therapy and increased mortality. Amphotericin B-based regimens remain the cornerstone of induction therapy for cryptococcosis [10,30]. However, this association should not be interpreted as evidence that a shorter treatment duration itself increased the risk of death. Rather, patients with severe disease were more likely to die before completing the planned course of L-AmB therapy, resulting in an apparently shorter treatment duration among non-survivors. Therefore, this finding most likely reflects reverse causality and is also susceptible to immortal time bias. Consequently, treatment duration should be interpreted as a marker of early mortality rather than an independent determinant of poor outcome.

In addition to these factors independently associated with one-year mortality, several clinical and laboratory parameters were associated with mortality in univariate analyses. A longer duration of immunosuppression was significantly more common among non-survivors, consistent with previous studies indicating that cumulative immune dysfunction increases susceptibility to severe infections [31]. Furthermore, the use of immunosuppressive therapies, particularly in non-HIV patients, was associated with worse outcomes, aligning with findings that highlight increased mortality in non-HIV cryptococcosis populations [23,32].

Notably, HIV infection itself was not associated with mortality in our study, consistent with previous reports indicating that outcomes in cryptococcosis are increasingly driven by disease severity and treatment-related factors rather than HIV status alone [23,33]. This observation was further supported by a sensitivity analysis in which HIV status was forced into the multivariable Cox model without materially changing the observed associations. Although HIV-associated cryptococcosis has historically been viewed as the main driver of mortality, recent studies show comparable—or even worse—outcomes in non-HIV populations. Subgroup analyses revealed important differences between PLWH and non-HIV patients. CNS involvement was significantly more frequent in PLWH, in line with the classical presentation of cryptococcal meningitis in advanced HIV infection. In contrast, non-HIV patients were older and had more heterogeneous clinical presentations. Despite these distinctions, mortality rates were similar, reinforcing that cryptococcal infection is no longer confined to advanced HIV infection and may carry an equally poor prognosis in non-HIV populations. Among non-HIV patients, adverse outcomes may be influenced by delayed diagnosis, heterogeneous underlying conditions, and low clinical suspicion. Indeed, some studies suggest that these patients present later in the disease course, which may contribute to worse outcomes [6]. Overall, these findings underscore the need for increased awareness of cryptococcal infections across diverse patient populations, regardless of HIV status.

Serum albumin was independently associated with improved survival in our study, highlighting its potential as a simple and clinically relevant prognostic marker in cryptococcal infections. Hypoalbuminemia has been linked to increased mortality across a range of infectious diseases and critical illness, reflecting both systemic inflammation and poor nutritional status [34]. Although it has been identified as an independent predictor of mortality in some cryptococcosis cohorts [35,36], this association has not been consistently observed. From a clinical perspective, serum albumin may reflect multiple pathophysiological processes, including systemic inflammation, capillary leakage, altered drug pharmacokinetics, and underlying nutritional status, all of which can contribute to adverse outcomes in cryptococcosis. Given its wide availability and low cost, serum albumin may represent a practical marker for early risk stratification, particularly in settings where access to advanced diagnostic resources is limited.

Laboratory findings also provided important prognostic insights. Non-survivors had significantly higher NLR, CRP, and ESR, along with lower lymphocyte counts. Elevated NLR is a marker of dysregulated systemic inflammatory responses and has been associated with poor outcomes in bacterial sepsis, invasive fungal infections, and other infectious diseases. These findings suggest that routinely available laboratory parameters may contribute to early clinical risk assessment. An increased fungal burden may contribute to exaggerated innate immune activation. Our study also highlights the role of nutritional status and systemic inflammation in disease prognosis.

The low rate of flucytosine use (5%) reflects limited access in our setting and may have contributed to the high mortality observed, given the established survival benefit of combination antifungal therapy [33]. The limited availability of flucytosine in Türkiye results from a combination of factors, including the lack of a commercial license for the drug on the Turkish market, global production and supply chain disruptions, and low market demand, given that it is a specialized drug used in rare cases [37]. A key strength of our study is that it reflects real-world clinical practice in a middle-income country, where access to flucytosine is limited. However, this limitation should be considered when interpreting our treatment outcomes, as it restricted the implementation of internationally recommended induction combination therapy and may limit the direct comparability of our findings with cohorts from settings where flucytosine is routinely available.

*P. laurentii* accounted for 8.7% of isolates in our cohort, a proportion that appears higher than that reported in most published series. This finding should be interpreted with caution, as species identification methods and taxonomic classification have evolved considerably over the study period. Recent reviews have highlighted the increasing recognition of rare basidiomycetous yeasts, including *Papiliotrema* species, as opportunistic pathogens in IC hosts, although their epidemiology remains incompletely defined [38]. Because of their distinct taxonomy and epidemiology, we performed a sensitivity analysis excluding *P. laurentii* cases. The results remained consistent, supporting the robustness of the primary findings. Because of the limited number of *P. laurentii* cases, species-specific analyses were not feasible. Dedicated studies focusing on *P. laurentii* infections are warranted to better define their epidemiology, clinical characteristics, and optimal management.

This study has several limitations. Its retrospective multicenter design introduces inherent limitations, including heterogeneity in clinical management across participating centers. Variability across centers in diagnostic and treatment approaches may have influenced outcomes. The relatively small sample size may limit the generalizability of our findings. In addition, important prognostic variables, including intracranial opening pressure, quantitative measures of fungal burden, and cryptococcal antigen (CrAg) titers, were not consistently available across centers. Although culture positivity was more frequently observed among non-survivors and may indirectly reflect a higher fungal burden, culture positivity is only a surrogate marker and does not directly quantify fungal burden. Furthermore, it may be influenced by factors such as specimen quality, the timing of sampling, and prior antifungal therapy. Therefore, this observation should be interpreted with caution. Another limitation of this study is that, because of its retrospective multicenter cohort design, population-based incidence rates of cryptococcal infections could not be calculated, as reliable denominator data for the general population and the HIV population served by the participating centers were not available.

Rather than identifying novel prognostic factors, our study provides multicenter real-world data on the clinical presentation, management, and outcomes of cryptococcal infections in a middle-income setting where access to recommended antifungal therapies remains limited. These findings complement the existing literature and contribute region-specific evidence from routine clinical practice.

## 5. Conclusions

In conclusion, altered consciousness, disseminated infection, and advanced age were independently associated with one-year mortality, whereas higher serum albumin levels were associated with improved survival in patients with cryptococcal infections. Although clinical presentations differ between PLWH and non-HIV patients, mortality rates were comparable, underscoring the need for heightened clinical awareness across diverse patient populations. This multicenter study provides real-world evidence from a middle-income setting and may help inform future research and healthcare planning in similar resource-constrained settings.

## Figures and Tables

**Table 1:** Baseline demographic and clinical characteristics of patients with cryptococcal infection according to survival status.

	All Patients (*n* = 92)	Non-Survivors (*n* = 43)	Survivors (*n* = 49)	*p* Value
Age	46 ± 16	51 ± 18	42 ± 13	0.007
Female gender	25 (27.1%)	17 (39.5%)	8 (16.3%)	0.013
History of bird feeding	11 (12%)	2 (4.6%)	9 (18.3%)	0.047
Diagnosis in 2020 and beyond	65 (70.7%)	28 (65.1%)	37 (75.5%)	0.275
CCI score	5.2 ± 3.5	5.3 ± 3.3	5.1 ± 3.7	0.627
Immunosuppression	80 (87%)	40 (93%)	40 (81.6%)	0.106
HIV infection	52 (56.5%)	22 (51.1%)	30 (61.2%)	0.331
Non-HIV immunosuppression	28 (30.4%)	18 (41.8%)	10 (20.4%)	0.224
- Hematological malignancy	10 (10.9%)	8 (18.6%)	2 (4%)	
- Solid organ malignancy	10 (10.9%)	6 (13.9%)	4 (8.1%)	
- Transplantation	4 (4.3%)	2 (4.6%)	2 (4%)	
- Rheumatological disease	6 (6.5%)	2 (4.6%)	4 (8.1%)	
Duration of immunosuppression (month)	26 ± 37	34 ± 42	18 ± 30	0.001
Immunosuppressive drug usage	29 (31.5%)	18 (41.8%)	11 (22.4%)	0.046
Steroid usage	15 (16.3%)	9 (20.9%)	6 (12.2%)	0.261
Other drug usage	23 (25%)	14 (32.5%)	9 (18.3%)	0.117
Intravenous drug usage	14 (15.2%)	6 (13.9%)	8 (16.3%)	0.951
Other opportunistic infection	34 (37%)	16 (37.2%)	18 (36.7%)	0.962
Symptoms				
Fever > 37.7 °C	61 (66.3%)	34 (79%)	27 (55.1%)	0.015
Headache	54 (58.7%)	26 (60.4%)	28 (57.1%)	0.747
Neck stiffness	25 (27.2%)	15 (34.8%)	10 (20.4%)	0.119
Nausea/vomiting	39 (42.4%)	17 (39.5%)	22 (44.8%)	0.604
Altered consciousness	40 (43.5%)	28 (65.1%)	12 (24.4%)	<0.001
Cranial nerve involvement	17 (18.5%)	9 (20.9%)	8 (16.3%)	0.570
Focal neurological sign	22 (23.9%)	12 (27.9%)	10 (20.4%)	0.400
Seizure	16 (17.4%)	12 (27.9%)	4 (8.1%)	0.013
Increased intracranial pressure	25 (27.2%)	12 (27.9%)	13 (26.5%)	0.882
Cough	38 (41.3%)	19 (44.1%)	19 (38.7%)	0.599
Dyspnea	32 (34.8%)	16 (37.2%)	16 (32.6%)	0.647
Tachypnea	28 (30.4%)	15 (34.8%)	13 (26.5%)	0.385
Skin lesion	16 (17.4%)	6 (13.9%)	10 (20.4%)	0.415
Duration of symptoms (day)	50 ± 80	40 ± 72	58 ± 86	0.145
GCS score	12.8 ± 2.9	11.4 ± 3.4	14 ± 1.6	<0.001
Infection involvement type				
CNS involvement	59 (64.1%)	27 (62.7%)	32 (65.3%)	0.802
Lung involvement	26 (28.3%)	14 (32.5%)	12 (24.4%)	0.391
Dissemination	25 (27.2%)	17 (39.5%)	8 (16.3%)	0.013
Skin involvement	11 (12%)	2 (4.6%)	9 (18.3%)	0.043

CCI: Charlson comorbidity index, GCS: Glasgow Coma Scale, CNS: Central nervous system. For categorical variables, *n* (%); for continuous variables, mean ± SD values were given.

**Table 2:** Baseline laboratory and treatment characteristics of patients with cryptococcal infection according to survival status.

	All Patients (*n* = 92)	Non-Survivors (*n* = 43)	Survivors (*n* = 49)	*p* Value
Culture positivity	69 (75%)	36 (83.7%)	33 (67.3%)	0.047
*Cryptococcus neoformans*	61 (66.3%)	34 (79%)	27 (55.1%)	
*Papiliotrema laurentii*	8 (8.7%)	2 (4.6%)	6 (12.2%)	
Laboratory results				
Leukocyte (cell/µL)	7983 ± 6539	7869 ± 4896	8083 ± 7751	0.460
Neutrophil (cell/µL)	6776 ± 6628	7083 ± 6029	6506 ± 7164	0.210
Lymphocyte (cell/µL)	928 ± 913	737 ± 734	1095 ± 1023	0.015
NLR	15.5 ± 25	24.1 ± 34	7.8 ± 6.8	0.004
Hemoglobin (g/dL)	11 ± 2.4	10.1 ± 2.1	11.8 ± 2.4	<0.001
Platelet (cell/µL)	210,722 ± 110,500	179,294 ± 117,911	238,302 ± 96,551	0.010
CRP (mg/L)	45 ± 56	67 ± 63	25 ± 41	<0.001
ESR (mm/h)	55 ± 29	63 ± 29	46 ± 27	0.004
AST (U/L)	36 ± 35	35 ± 25	37 ± 42	0.493
ALT (U/L)	34 ± 41	30 ± 29	37 ± 49	0.436
Creatinine (mg/dL)	0.9 ± 0.7	1.1 ± 0.8	0.9 ± 0.6	0.182
Albumin (g/dL)	3.1 ± 0.7	2.8 ± 0.6	3.3 ± 0.7	<0.001
CD4+ T lymphocyte (cell/µL)	75 ± 99	58 ± 66	88 ± 117	0.541
CD8+ T lymphocyte (cell/µL)	488 ± 358	475 ± 400	497 ± 330	0.831
CD4/CD8 ratio	0.15 ± 0.18	0.13 ± 0.12	0.17 ± 0.21	0.656
HIV RNA (copy/mL)	2,044,330 ± 5,698,040	3,578,323 ± 8,483,807	919,404 ± 1,372,680	0.176
Pathological cranial MRI	41/59 (69.5%)	20/27 (74%)	21/32 (65%)	0.124
Hydrocephalus	16/59 (27.1%)	10/27 (37%)	6/32 (18.7%)	0.164
Cerebral infarction	20/59 (33.8%)	10/27 (37%)	10/32 (31.2%)	0.741
Cerebral edema	9/59 (15.2%)	5/27 (18.5%)	4/32 (12.5%)	0.417
Cryptococcoma	16/59 (27.1%)	6/27 (22.2%)	10/32 (31.2%)	0.415
Treatment characteristics				
L-AmB usage	74 (80.4%)	36 (83.7%)	38 (77.5%)	0.457
L-AmB, duration of therapy (day)	20 ± 14	13 ± 8	26 ± 14	<0.001
FLU usage	84 (91.3%)	35 (81.3%)	49 (100%)	0.002
FLU, duration of therapy (day)	170 ± 285	67 ± 141	244 ± 336	<0.001
Flucytosine	5 (5.4%)	2 (4.6%)	3 (6.1%)	0.562
Flucytosine, duration of therapy (day)	21 ± 7	22 ± 11	20 ± 7	0.819
L-AmB + FLU usage	72 (78.3%)	34 (79%)	38 (77.5%)	0.860
Surgery	10 (10.9%)	5 (11.6%)	5 (10.2%)	0.544
Therapeutic lumbar puncture	23/59 (38.9%)	10/27 (37%)	13/32 (40%)	0.717
Total duration of therapy (day)	164 ± 284	60 ± 135	256 ± 344	<0.001
Duration of ward hospitalization (day)	37 ± 66	32 ± 90	42 ± 33	<0.001
ICU admission	45 (48.9%)	32 (74.4%)	13 (26.5%)	<0.001
Duration of ICU stay (day)	17 ± 19	16 ± 19	18 ± 19	0.940
Unintended consequences				
IRIS	4 (4.3%)	3 (6.9%)	1 (2%)	0.261
Relapse	7 (7.6%)	0	7 (14.2%)	0.010
Sequelae	12 (13%)	0	12 (24.4%)	<0.001

NLR: Neutrophil/lymphocyte ratio, CRP: C-reactive protein, ESR: Erythrocyte sedimentation rate, AST: Aspartate aminotransferase, ALT: Alanine aminotransferase, MRI: Magnetic resonance imaging, L-AmB: Liposomal amphotericin B, FLU: Fluconazole, IRIS: Immune reconstitution inflammatory syndrome, ICU: Intensive care unit. For categorical variables, *n* (%); for continuous variables, mean ± SD values were given.

**Table 3:** Baseline characteristics of *Papiliotrema laurentii* infections.

	Case 1	Case 2	Case 3	Case 4	Case 5	Case 6	Case 7	Case 8
Age	40	32	66	77	31	43	50	65
Gender	F	F	M	M	M	M	M	F
Charlson comorbidity index	2	4	12	3	9	4	0	11
Immunosuppression	+	+	+	+	+	−	+	−
HIV infection	−	−	−	−	−	−	−	−
Duration of immunosuppression (month)	4	3	1	1	2	−	3	−
Pathogen identification method	MALDI-TOF MS	MALDI-TOF MS	MALDI-TOF MS	MALDI-TOF MS	MALDI-TOF MS	MALDI-TOF MS	MALDI-TOF MS	MALDI-TOF MS
Infection involvement type	Lung infection	Lung infection	Lung infection	Lung infection	Lung infection	Skin İnfection	Skin infection	Urinary infection
ICU admission	−	−	−	+	−	+	−	−
Mortality	−	−	−	+	+	−	−	−
Relapse	−	−	−	−	−	−	−	−
Sequelae	−	−	−	−	−	−	−	−

F: Female, M: Male, ICU: Intensive care unit.

**Table 4:** Univariate and multivariable Cox proportional hazards regression analyses for one-year mortality.

Variable	Crude HR (95% CI)	*p* Value	Adjusted HR (95% CI)	*p* Value
Age (per year)	1.031 (1.011–1.051)	0.002	1.025 (1.006–1.045)	0.010
Disseminated infection	2.228 (1.206–4.114)	0.010	2.155 (1.156–4.016)	0.016
Altered consciousness *	3.546 (1.881–6.685)	<0.001	3.110 (1.624–5.956)	<0.001
Albumin (per g/dL)	0.487 (0.317–0.747)	0.001	0.532 (0.328–0.861)	0.010

HR: Hazard ratio; CI: Confidence interval. HR < 1 indicates an inverse association with mortality. * Sensitivity analysis using a GCS threshold of ≤13 showed similar results (crude HR 3.343, 95% CI 1.811–6.172, *p* < 0.001; adjusted HR 2.813, 95% CI 1.501–5.273, *p* = 0.001).

## Data Availability

The data presented in this study are available on request from the corresponding author due to privacy.

## Supplementary Materials

The following supporting information can be downloaded at https://www.mdpi.com/article/10.3390/jof12080545/s1. Table S1: Sensitivity analyses of the multivariable Cox proportional hazards model for one-year mortality. Table S2: Baseline demographic and clinical characteristics of patients with cryptococcal infection according to HIV status. Table S3: Baseline laboratory and treatment characteristics of patients with cryptococcal infection according to HIV status.

## References

[1] Perfect J.R., Boulware D.R., Blaser M.J., Cohen J.I., Holland S.M. (2026). Cryptococcosis (*Cryptococcus neoformans* and *Cryptococcus gattii*). Mandell, Douglas, and Bennett’s Principles and Practice of Infectious Diseases.

[2] Smith N., Sehring M., Chambers J., Patel P. (2017). Perspectives on non-neoformans cryptococcal opportunistic infections. J. Community Hosp. Intern. Med. Perspect..

[3] Rajasingham R., Smith R.M., Park B.J., Jarvis J.N., Govender N.P., Chiller T.M., Denning D.W., Loyse A., Boulware D.R. (2017). Global burden of disease of HIV-associated cryptococcal meningitis: An updated analysis. Lancet Infect. Dis..

[4] Sloan D.J., Parris V. (2014). Cryptococcal meningitis: Epidemiology and therapeutic options. Clin. Epidemiol..

[5] Brizendine K.D., Baddley J.W., Pappas P.G. (2013). Predictors of mortality and differences in clinical features among patients with cryptococcosis according to immune status. PLoS ONE.

[6] Namie H., Takazono T., Hidaka Y., Morimoto S., Ito Y., Nakada N., Ashizawa N., Hirayama T., Takeda K., Iwanaga N. (2024). The prognostic factors for cryptococcal meningitis in non-HIV patients: An observational nationwide study. Mycoses.

[7] Tao Z., Pu Q., Shen Y., Zhang S., Wang C., Ju Z., Jin Y., Zhu X., Weng Y. (2024). Clinical characteristics and prognostic factors of pulmonary and extrapulmonary cryptococcosis. BMC Infect. Dis..

[8] Gu L., Lin J., Li A., Yue J., Wen W., Liu W., Lin Q., Chen X., Chen X., Wu J. (2026). Clinical spectrum, immune status, and prognostic factors of cryptococcosis: Insights from a large multicenter ambispective cohort study in southeastern China. Infect. Dis. Poverty.

[9] Lu C.H., Chang W.N., Chang H.W., Chuang Y.C. (1999). The Prognostic factors of cryptococcal meningitis in HIV-negative patients. J. Hosp. Infect..

[10] World Health Organization (2022). Guidelines for Diagnosing, Preventing and Managing Cryptococcal Disease Among Adults, Adolescents and Children Living with HIV.

[11] Wu L., Fu X., Pütz B., Zhang R., Liu L., Song W., Weng L., Shao Y., Zheng Z., Xun J. (2024). Comprehensive risk factor predictions for 3-year survival among HIV-associated and disseminated cryptococcosis involving lungs and central nervous system. Infection.

[12] Liu J., Liu J., Xu L., Yang L., Su X., Lu Y., Li D., Li Z., Hoi C.P., Jiang Y. (2025). Evaluating clinical outcomes in non-HIV associated cryptococcal meningitis with negative CSF culture using microscopic quantitative cryptococcal counting. Diagn. Microbiol. Infect. Dis..

[13] Chang C.C., Harrison T.S., Bicanic T.A., Chayakulkeeree M., Sorrell T.C., Warris A., Hagen F., Spec A., Oladele R., Govender N.P. (2024). Global guideline for the diagnosis and management of cryptococcosis: An initiative of the ECMM and ISHAM in cooperation with the ASM. Lancet Infect. Dis..

[14] Hakyemez I.N., Erdem H., Beraud G., Lurdes M., Silva-Pinto A., Alexandru C., Bishop B., Mangani F., Argemi X., Poinot M. (2018). Prediction of unfavorable outcomes in cryptococcal meningitis: Results of the multicenter Infectious Diseases International Research Initiative (ID-IRI) cryptococcal meningitis study. Eur. J. Clin. Microbiol. Infect. Dis..

[15] Haddow L.J., Colebunders R., Meintjes G., Lawn S.D., Elliott J., Manabe Y.C., Bohjanen P.R., Sungkanuparph S., Easterbrook P.J., French M.A. (2010). Cryptococcal immune reconstitution inflammatory syndrome in HIV-1-infected individuals: Proposed clinical case definitions. Lancet Infect. Dis..

[16] Park B.J., Wannemuehler K.A., Marston B.J., Govender N., Pappas P.G., Chiller T.M. (2009). Estimation of the current global burden of cryptococcal meningitis among persons living with HIV/AIDS. AIDS.

[17] Coussement J., Heath C.H., Roberts M.B., Lane R.J., Spleman T., Smibert O.C., Longhitano A., Morrissey C.O., Nield B., Tripathy M. (2025). Management, Outcomes, and Predictors of Mortality of *Cryptococcus* Infection in Patients Without HIV: A Multicenter Study in 46 Hospitals in Australia and New Zealand. Clin. Infect. Dis..

[18] Fiorica G., Cerver-Hernandez M.E., Pirofski L.A., Hemmige V.S., Yoon H. (2025). High Mortality and Associated Risk Factors in Kidney Transplant Recipients with Cryptococcosis-A Nationwide Cohort Study Over a Decade Using USRDS Data. Open Forum Infect. Dis..

[19] Zhao Z., Song W., Liu L., Qi T., Wang Z., Tang Y., Sun J., Xu S., Yang J., Wang J. (2024). Trends in Clinico-Epidemiological Profile and Outcomes of Patients with HIV-Associated Cryptococcal Meningitis in Shanghai, China, 2013–2023. Viruses.

[20] Liu S., Chen W., Cheng F., Ye X., Pan N., Lu H. (2023). Clinical characteristics and prognostic factors of 60 patients with acquired immune deficiency syndrome combined with Cryptococcus neoformans. BMC Infect. Dis..

[21] Wang F., Wang Y., He J., Cheng Z., Wu S., Wang M., Niu T. (2022). Clinical Characteristics and Risk Factors for Mortality in Cryptococcal Meningitis: Evidence From a Cohort Study. Front. Neurol..

[22] Person A.K., Crabtree-Ramirez B., Kim A., Veloso V., Maruri F., Wandeler G., Fox M., Moore R., Gill M.J., Imran D. (2023). Cryptococcal Meningitis and Clinical Outcomes in Persons With Human Immunodeficiency Virus: A Global View. Clin. Infect. Dis..

[23] Bratton E.W., El Husseini N., Chastain C.A., Lee M.S., Poole C., Stürmer T., Juliano J.J., Weber D.J., Perfect J.R. (2012). Comparison and temporal trends of three groups with cryptococcosis: HIV-infected, solid organ transplant, and HIV-negative/non-transplant. PLoS ONE.

[24] Lin Y.Y., Shiau S., Fang C.T. (2015). Risk Factors for Invasive *Cryptococcus neoformans* Diseases: A Case-Control Study. PLoS ONE.

[25] Bicanic T., Harrison T.S. (2005). Cryptococcal meningitis. Br. Med. Bull..

[26] Jarvis J.N., Bicanic T., Loyse A., Namarika D., Jackson A., Nussbaum J.C., Longley N., Muzoora C., Phulusa J., Taseera K. (2014). Determinants of mortality in a combined cohort of 501 patients with HIV-associated Cryptococcal meningitis: Implications for improving outcomes. Clin. Infect. Dis..

[27] Xie H., Luo P., Li Z., Li R., Sun H., Wu D. (2017). Continuous intrathecal administration of liposomal amphotericin B for treatment of refractory Cryptococcus neoformans encephalitis: A case report. Exp. Ther. Med..

[28] Alvarez-Uria G., Midde M., Pakam R., Yalla P.S., Naik P.K., Reddy R. (2015). Short-Course Induction Treatment with Intrathecal Amphotericin B Lipid Emulsion for HIV Infected Patients with Cryptococcal Meningitis. J. Trop. Med..

[29] Soraci L., Beccacece A., Princiotto M., Saverda E.V., Gambuzza M.E., Aguennouz M.H., Corsonello A., Luciani F., Muglia L., Filicetti E. (2024). The emerging links between immunosenescence in innate immune system and neurocryptococcosis. Front. Immunol..

[30] Perfect J.R., Dismukes W.E., Dromer F., Goldman D.L., Graybill J.R., Hamill R.J., Harrison T.S., Larsen R.A., Lortholary O., Nguyen M.-H. (2010). Clinical Practice Guidelines for the Management of Cryptococcal Disease: 2010 Update by the Infectious Diseases Society of America. Clin. Infect. Dis..

[31] Williamson P.R., Jarvis J.N., Panackal A.A., Fisher M.C., Molloy S.F., Loyse A., Harrison T.S. (2017). Cryptococcal meningitis: Epidemiology, immunology, diagnosis and therapy. Nat. Rev. Neurol..

[32] Pappas P.G. (2013). Cryptococcal infections in non-HIV-infected patients. Trans. Am. Clin. Climatol. Assoc..

[33] Molloy S.F., Kanyama C., Heyderman R.S., Loyse A., Kouanfack C., Chanda D., Mfinanga S., Temfack E., Lakhi S., Lesikari S. (2018). Antifungal Combinations for Treatment of Cryptococcal Meningitis in Africa. N. Engl. J. Med..

[34] Vincent J.L., Dubois M.J., Navickis R.J., Wilkes M.M. (2003). Hypoalbuminemia in acute ıllness: Is there a rationale for ıntervention?. Ann. Surg..

[35] Zhao H., Zhou M., Zheng Q., Zhu M., Yang Z., Hu C., Xu L. (2021). Clinical features and Outcomes of Cryptococcemia patients with and without HIV infection. Mycoses.

[36] Zhang F., Zhou Y., Tang X., Li M. (2023). Identification of risk factors for disseminated cryptococcosis in non-HİV patients: A retrospective analysis. Eur. J. Med. Res..

[37] Sigera L.S.M., Denning D.W. (2023). Flucytosine and its clinical use. Ther. Adv. Infect. Dis..

[38] Morales Lopez S.E., Garcia-Effron G. (2021). Infections due to Rare *Cryptococcus* Species. A Literature Review. J. Fungi.

