# Supplementary material for: Clinical Characteristics and Predictors of One-Year Mortality in HIV and Non-HIV Patients with Cryptococcal Infections in a Middle-Income Setting: A Multicenter Cohort Study
Source: J Fungi (Basel). 2026 Jul 23;12(8):545. doi: 10.3390/jof12080545 (PMC13514301; doi:10.3390/jof12080545)
Supplement: Supplementary file 1 [file jof-12-00545-s001.zip › jof-4418269-supplementary.pdf]

Supplementary Table S1- Sensitivity analyses of the multivariable Cox proportional hazards model for one-year mortality

Variable	**Original model** (n=92)		**Excluding *P. laurentii*** (n=84)		**Excluding *P. laurentii* + HIV forced into model** (n=84)	
	**Adjusted HR (95% CI)**	***p* value**	**Adjusted HR (95% CI)**	***p* value**	**Adjusted HR (95% CI)**	***p* value**
Age (per year)	1.025 (1.006–1.045)	0.010	1.023 (1.003-1.043)	0.025	1.021 (1.001-1.043)	0.048
Disseminated infection	2.155 (1.156–4.016)	0.016	2.293 (1.212-4.337)	0.011	2.281 (1.204-4.322)	0.011
Altered consciousness	3.110 (1.624–5.956)	<0.001	3.280 (1.653-6.509)	<0.001	3.312 (1.667-6.581)	<0.001
Albumin (per g/dL)	0.532 (0.328–0.861)	0.010	0.516 (0.313-0.853)	0.010	0.523 (0.315-0.868)	0.012
HIV	-	-	-	-	0.865 (0.436-1.716)	0.678

HR: Hazard ratio, CI: Confidence interval, HR < 1 indicates an inverse association with mortality.

Supplementary Table S2- Baseline demographical and clinical characteristics of patients with cryptococcal infection according to HIV status

	**All patients** (n=92)	**HIV cases** (n=52)	**Non-HIV cases** (n=40)	*p* **value**
**Age**	46 ± 16	41 ± 11	56 ± 17	**0.002**
**Female gender**	25 (27.2%)	7 (13.5%)	18 (45%)	**<0.001**
**CCI score**	5.2 ± 3.5	6.2 ± 3.5	3.9 ± 2.8	**<0.001**
**Immunosuppression**	80 (87%)	52 (100%)	28 (70%)	**<0.001**
**Duration of immunosuppression** (month)	26 ± 37	17 ± 15	41 ± 55	0.108
**Immunosuppressive drug usage**	29 (31.5%)	7 (13.5%)	22 (55%)	**<0.001**
**Intravenous drug usage**	14 (15.2%)	11 (21.2%)	3 (7.5%)	**0.026**
**Other opportunistic infection**	34 (37%)	29 (55.8%)	5 (12.5%)	**<0.001**
**Symptoms**				
Fever >37.7°C	61 (66.3%)	36 (69.2%)	25 (62.5%)	0.498
Headache	54 (58.7%)	38 (73.1%)	16 (40%)	**<0.001**
Neck stiffness	25 (27.2%)	17 (32.7%)	8 (20%)	0.175
Nausea/vomiting	39 (42.4%)	21 (40.4%)	18 (45%)	0.657
Altered consciousness	40 (43.5%)	25 (48.1%)	15 (37.5%)	0.310
Cranial nerve involvement	17 (18.5%)	8 (15.4%)	9 (22.5%)	0.383
Focal neurological sign	22 (23.9%)	14 (26.9%)	8 (20%)	0.440
Seizure	16 (17.4%)	12 (23.1%)	4 (10%)	0.101
Increased intracranial pressure	25 (27.2%)	19 (36.5%)	6 (15%)	**0.021**
**GCS score**	12.8 ± 2.9	13 ± 2.8	12.5 ± 3.3	0.848
**Infection involvement type**				
CNS involvement	59 (64.1%)	41 (78.8%)	18 (45%)	**<0.001**
Lung involvement	26 (28.3%)	15 (28.8 %)	11 (27.5 %)	0.887
Dissemination	25 (27.2%)	14 (26.9%)	11 (27.5%)	0.951
Skin involvement	11 (12%)	6 (11.5%)	5 (12.5%)	0.568

CCI: Charlson comorbidity index, GCS: Glasgow coma scale, CNS: Central nervous system.

For categorical variables, n (%); for continuous variables, mean ± SD values are given.

Supplementary Table S3- Baseline laboratory and treatment characteristics of patients with cryptococcal infection according to HIV status

	All patients (n=92)	HIV cases (n=52)	Non-HIV cases (n=40)	*p* value
**Culture positivity**	69 (75%)	35 (67.3%)	34 (85%)	0.073
**Serum CrAg test positivity**	34 (37%)	25 (48.1%)	9 (22.5%)	**0.021**
**CSF CrAg test positivity**	19 (20.7%)	13 (25%)	6 (15%)	**0.016**
**Laboratory results**				
Leukocyte (cell/µL)	7983 ± 6539	5555 ± 3008	11696 ± 9128	**<0.001**
Neutrophil (cell/µL)	6776 ± 6628	4423 ± 3022	10557 ± 9417	**<0.001**
Lymphocyte (cell/µL)	928 ± 913	806 ± 658	995 ± 1040	0.375
NLR	15.5 ± 25	13.8 ± 25.8	20.8 ± 27	0.101
Hemoglobin (g/dL)	11 ± 2.4	10.7 ± 2	11.3 ± 2.8	0.248
Platelet (cell/µL)	210722 ± 110500	213815 ± 106035	206701 ± 117295	0.761
CRP (mg/L)	45 ± 56	38 ± 50	52 ± 63	0.212
ESR (mm/h)	55 ± 29	61 ± 29	46 ± 27	**0.015**
AST (U/L)	36 ± 35	35 ± 24	37 ± 45	0.121
ALT (U/L)	34 ± 41	31 ± 27	37 ± 54	0.282
Creatinine (mg/dL)	0.9 ± 0.7	0.8 ± 0.3	1.2 ± 0.9	0.304
Albumin (g/dL)	3.1 ± 0.7	3.1 ± 0.7	3 ± 0.7	0.623
**Pathological cranial MRI**	41/59 (69.5%)	25/41 (60.9%)	16/18 (88.8%)	0.604
Hydrocephalus	16/59 (27.1%)	9/41 (21.9%)	7/18 (38.8%)	0.981
Cerebral infarction	20/59 (33.8%)	16/41 (39%)	4/18 (22.2%)	**0.017**
Cerebral edema	9/59 (15.2%)	5/41 (12.1%)	4/18 (22.2%)	0.609
Cryptococcoma	16/59 (27.1%)	12/41 (29.2%)	4/18 (22.2%)	0.101
**Treatment characteristics**				
L-AmB usage	74 (80.4%)	46 (88.5%)	28 (70%)	**0.027**
Surgery	10 (10.9%)	6 (11.5%)	4 (10%)	0.545
Therapeutic lumbar puncture	23/59 (38.9%)	15/41 (36.5%)	8/18 (44.4%)	0.331
Total duration of therapy (day)	164 ± 284	208 ± 354	107 ± 106	0.056

**Duration of ward hospitalization** (day)	37 ± 66	47 ± 83	25 ± 29	**0.017**
**ICU admission**	45 (48.9%)	23 (44.2%)	22 (55%)	0.306
**Duration of ICU stay** (day)	17 ± 19	11 ± 11	23 ± 24	**0.037**
**Mortality**	43 (46.7%)	22 (42.3%)	21 (52.5%)	0.331
**Relapse**	7 (7.6%)	6 (11.5%)	1 (2.5%)	0.108
**Sequelae**	12 (13%)	6 (11.5%)	6 (15%)	0.625

CrAg: Cryptococcal antigen, CSF: Cerebrospinal fluid, NLR: Neutrophil/lymphocyte ratio, CRP: C-reactive protein, ESR: Erythrocyte sedimentation rate, AST: Aspartate aminotransferase, ALT: Alanine aminotransferase, MRI: Magnetic resonance imaging, L-AmB: Liposomal amphotericin B, FLU: Fluconazole, IRIS: Immune reconstitution inflammatory syndrome, ICU: Intensive care unit.

For categorical variables, n (%); for continuous variables, mean ± SD values are given.
